# Comparative study of antibacterial properties of polystyrene films with TiO*_x_* and Cu nanoparticles fabricated using cluster beam technique

**DOI:** 10.3762/bjnano.9.80

**Published:** 2018-03-12

**Authors:** Vladimir N Popok, Cesarino M Jeppesen, Peter Fojan, Anna Kuzminova, Jan Hanuš, Ondřej Kylián

**Affiliations:** 1 Department of Materials and Production, Aalborg University, Skjernvej 4A, 9220 Aalborg, Denmark; 2 Department of Macromolecular Physics, Charles University, V Holešovičkách 2, 18000 Prague 8, Czech Republic

**Keywords:** antibacterial properties, cluster beam technique, nanoparticles, polymers

## Abstract

**Background:** Antibacterial materials are of high importance for medicine, and for the production and conservation of food. Among these materials, polymer films with metal nanoparticles (NPs) are of considerable interest for many practical applications.

**Results:** The paper describes a novel approach for the formation of bactericidal polymer thin films (polystyrene in this case), produced by spin-coating, with Ti and Cu NPs deposited from cluster beams. Ti NPs are treated in three different ways in order to study different approaches for oxidation and, thus, efficiency in formation of the particles with semiconducting properties required for the catalytic formation of reactive oxygen species. Cu NPs are used as deposited. Partial NP embedding into polystyrene is realised in a controllable manner using thermal annealing in order to improve surface adhesion and make the particles resistant against wash-out. The formed composite films with TiO*_x_* and Cu species are tested as bactericidal media using *E.coli* bacteria as model microorganisms.

**Conclusion:** The obtained results show considerable efficiency in destroying the bacteria and a good possibility of multiple re-use of the same composite films making the suggested approach attractive for the cases requiring reusable polymer-based antibacterial media.

## Introduction

Certain metals, when present in excess, become destructive or lethal to organic cells. Because of these toxic properties the films and nanostructures of such metals or metal compounds are widely used as antibacterial and antimicrobial agents. Among them are Ag, Cu, Au, CuO, ZnO, Fe_3_O_4_, Al_2_O_3_ and TiO_2_, to name just a few [1–3]. They all exhibit bactericidal properties through mechanisms based on (i) metal ion selectivity (replacement of original metals leading to cellular dysfunction); (ii) metal reduction potential (generating or catalysing the formation of reactive oxygen species (ROS) damaging cellular proteins, lipids and DNA) and (iii) direct nanoparticle (NP) interaction with bacterial surfaces, which can lead to blocking of membrane transport channels and disturbing of electrochemical gradients [4]. Ag NPs are the most popular and most extensively studied pure metal NPs. However, Cu NPs are found to demonstrate higher toxicity for certain types of bacteria [1], and they are also cheaper compared to silver. These particles mainly show bactericidal effects through mechanisms (i) and (iii). Among the metal oxides, TiO_2_ is the best-known and most widely used semiconductor material with a wide band gap that under UV illumination generates electron–hole pairs affecting in a destructive way the cell components but rendering innocuous products (mechanism ii) [5–6]. Thus, both Cu and TiO_2_ nanoparticles are excellent candidates for the formation of composites with antibacterial and antimicrobial properties.

For more convenience in use and extended applications, NPs can be embedded into polymer films. Polymers have a clear advantage as cheap and plastic materials that can be easily fabricated in shapes and forms convenient for practical and simple use. Hence, metal–polymer composites have become very attractive, for example, in food packaging or the fabrication of consumables for medicine (for example, catheters for drug delivery) [7–8].

There are different ways for the formation of polymers with metal NPs. Particles can be synthesized in situ using the organic matrix as the reaction medium or ex situ by chemical or physical means and then incorporated into the matrix [4]. It is worth mentioning that the methods providing dispersion of NPs in the polymer bulk are not of high interest for bactericidal applications. Particles must be located at surfaces to be active agents. At the same time, adhesion of NPs should be considerably high in order to be resistant against washing out in procedures.

It has recently been shown that the deposition of metal NPs using the cluster beam technique is an efficient approach in the formation of polymer composite films with antibacterial properties. The composites can be formed by either incorporation of particles formed in a cluster source into polymer simultaneously synthesized by plasma-enhanced chemical vapour deposition, by coating the NPs with a thin overlay or by soft landing of clusters on ex situ fabricated films followed by thermal annealing facilitating the partial embedding of clusters into the polymer [9–15].

In the current work, both Ti and Cu clusters are produced using magnetron sputtering in special cluster sources and deposited on substrates coated by a thin layer of polystyrene (PS). The focus is on the optimization of the nanocomposite formation using the cluster beam technique and the material properties as well as on the comparison of bactericidal efficiency of the prepared composites with either TiO*_x_* or Cu NPs against *E.coli* bacteria as a model microorganism.

## Results and Discussion

### Morphology and composition of deposited clusters

**Figure 1:**
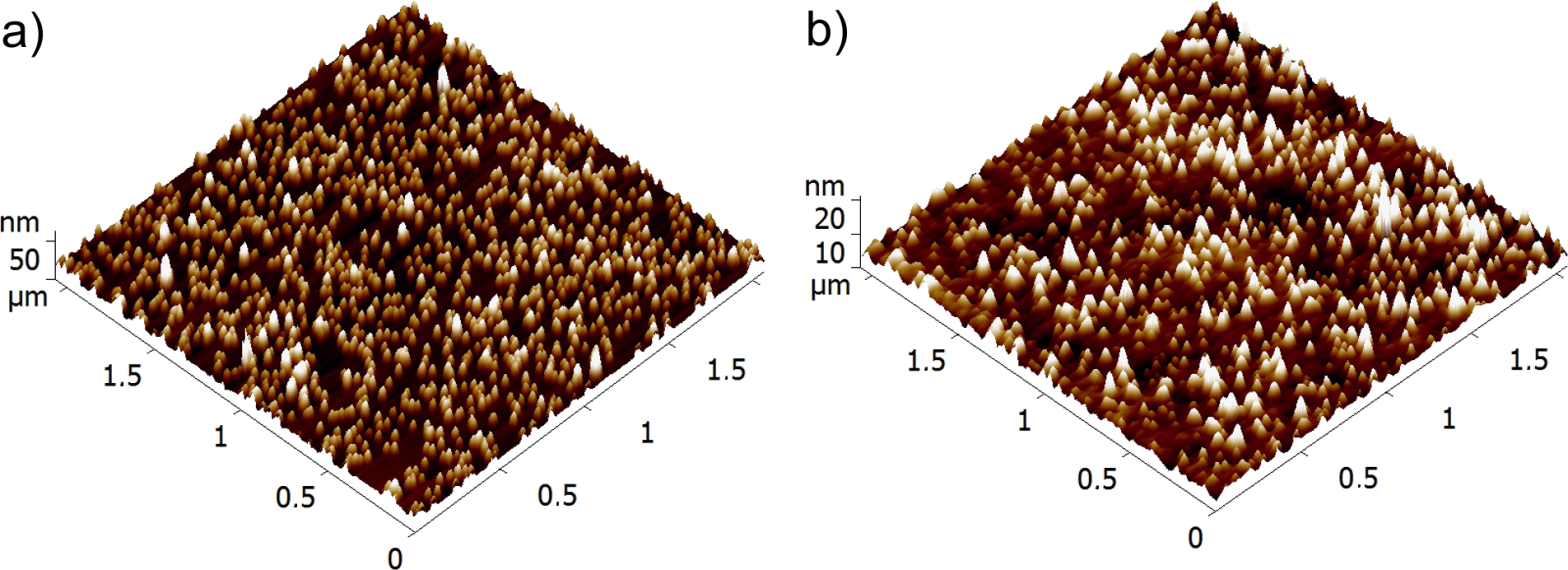
AFM images of (a) Cu clusters as-deposited on PS and (b) clusters after thermal annealing causing partial embedding of NPs.

**Figure 2:**
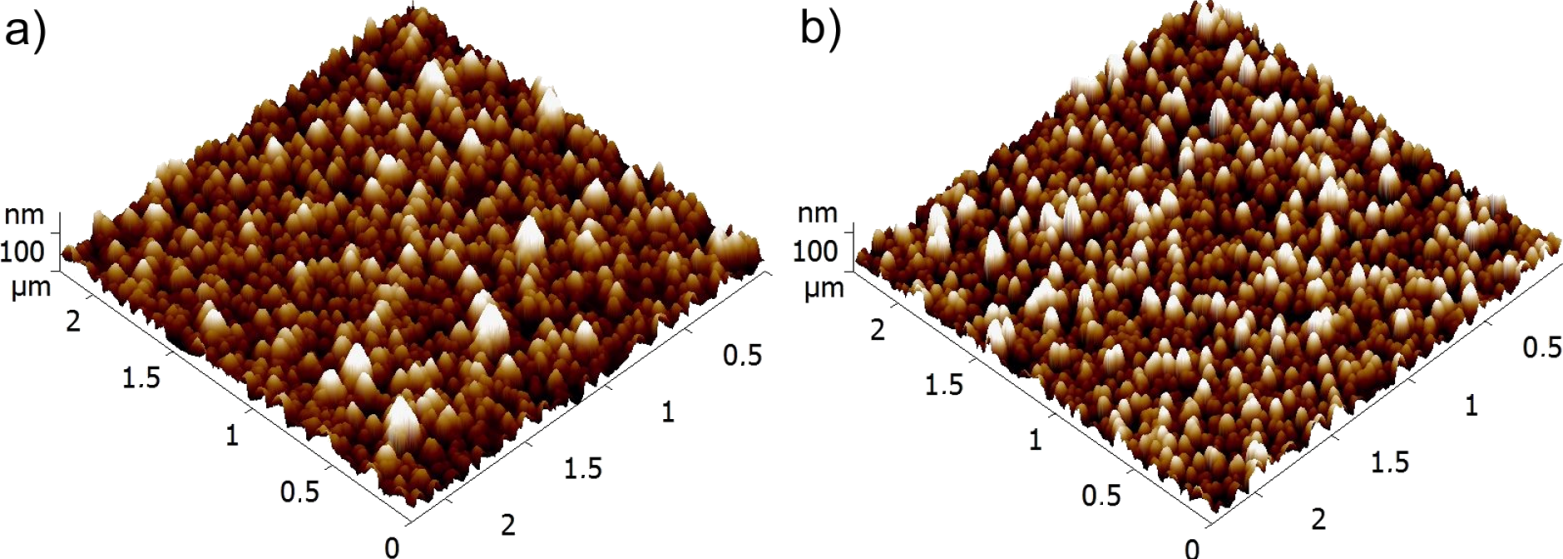
AFM images of sample type 2 (a) after Ti cluster deposition and annealing (120 °C for 5 min in ambient atmosphere) and (b) after use in antibacterial experiment and washing out the cells (5th cycle).

Copper cluster are deposited from the source under the conditions described in the Experimental section (see below). A typical atomic force microscopy (AFM) image with soft-landed Cu NPs on PS is shown in Figure 1a. Apart of a few higher bumps due to stacking of particles on top of each other, one can see an even height distribution because the Cu clusters are size-selected prior the deposition (see Experimental section). The annealing leads to the immersion of NPs into PS by about 30–50% of their diameter as can be seen in Figure 1b while comparing the height scales in panels (a) and (b). These images are given just for the illustration of the NP embedding. For the antibacterial tests, the samples with higher surface cluster coverage (slightly more than one monolayer of particles) are used. A very similar tendency for immersion is found for Ti NPs on PS. Therefore, the AFM images are not presented. The AFM measurements also show that the surface cluster density is not changed after the deposition of bacteria and the following washing of the samples over a few cycles of the experiments. Such example is presented in Figure 2 for Ti NPs.

**Figure 3:**
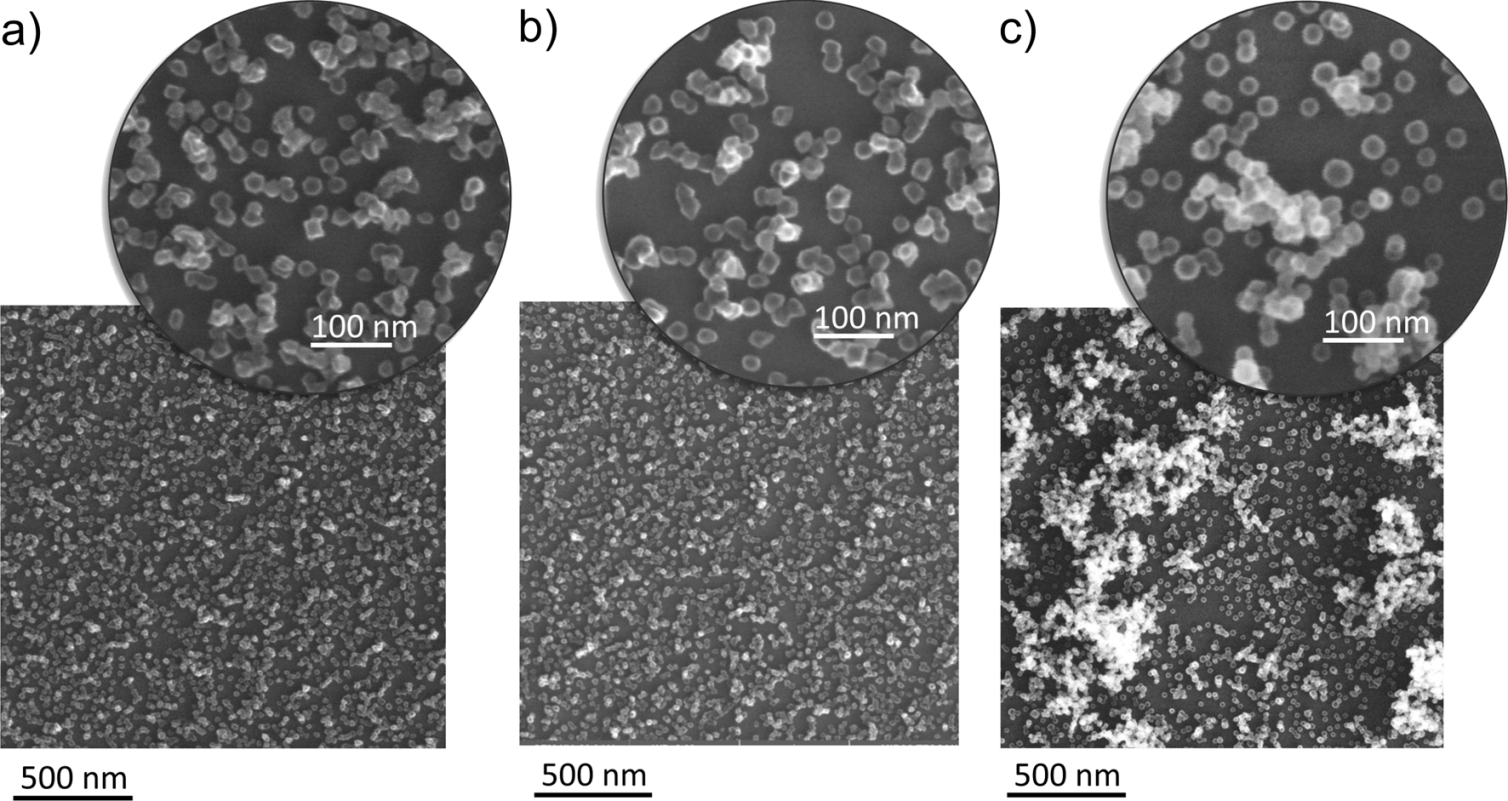
SEM images of samples of (a) type 1, (b) type 2 and (c) type 3. Round inserts show high-magnification images for every case.

Ti clusters produced by magnetron sputtering were either left untreated or oxidized in two different ways (see Experimental section for details) and denoted as “type 1”, “type 2” and “type 3”. Scanning electron microscopy (SEM) images of the Ti/TiO*_x_* NPs for all three types are shown in Figure 3. The particles of type 1 and 2 predominantly have polyhedral shapes while NPs of type 3 are more rounded. Mean sizes of the particles were measured from SEM images and found to be 18.9 ± 2.2, 20.3 ± 1.7 and 21.4 ± 2.1 nm for type 1, 2 and 3, respectively. Thus, the particle sizes are very similar for all three types. Scanning of larger areas shows that NPs of type 1 and 2 are homogeneously and randomly distributed across the surface while particles of type 3 tend to form aggregates (see Figure 3c). A comparison of SEM and AFM images obtained from particles deposited on bare Si and Si covered with PS films does not show any considerable difference allowing one to conclude that the presence of the thin polymer film does not affect the morphology.

**Figure 4:**
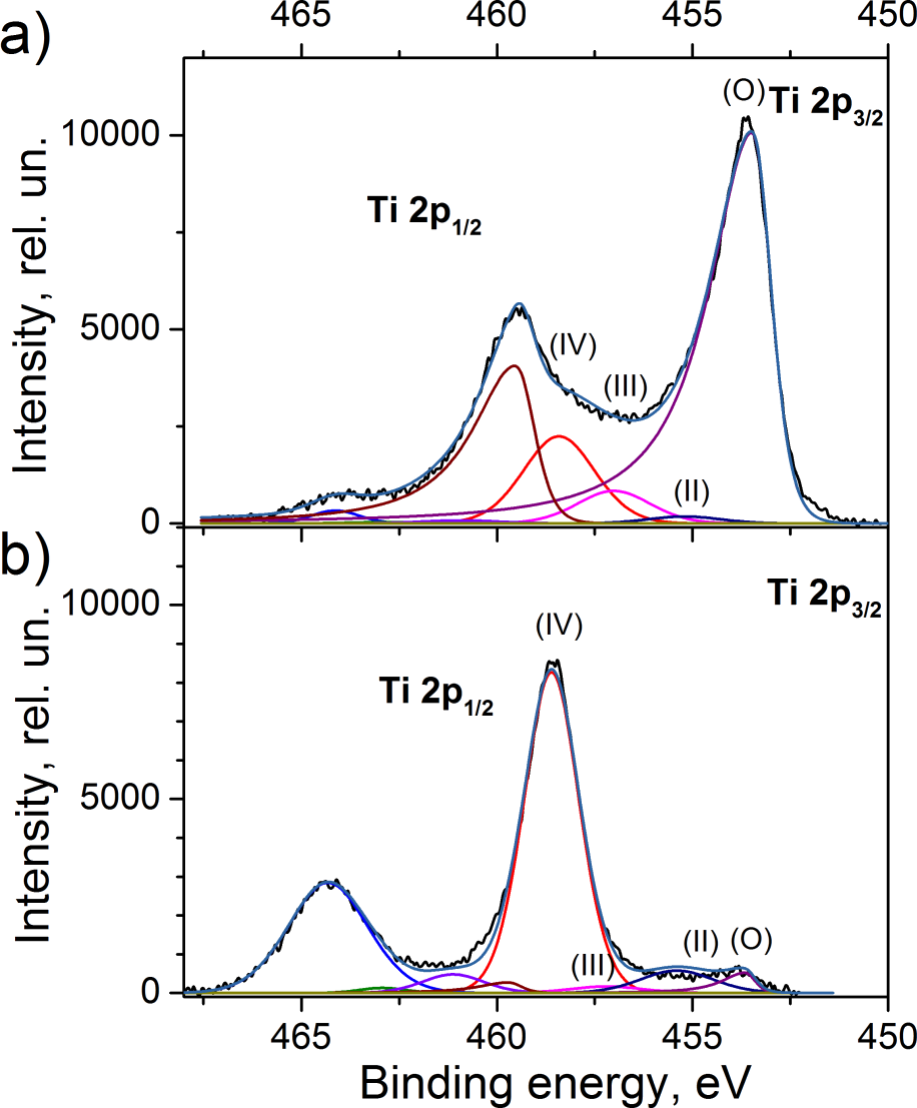
High-resolution XPS spectra of the Ti 2p peak of a) Ti clusters without oxygen treatment (type 1) and b) Ti clusters in-flight oxidized by RF plasma (type 3) measured immediately after the deposition. For better clarity, the Ti states are only presented for the Ti 2p_3/2_ peak.

Since Ti clusters were prepared in three different manners, they exhibit different compositions as confirmed by means of X-ray photoelectron spectroscopy (XPS). Ti NPs of type 1 immediately after the deposition demonstrate the peak at a binding energy of 453.7 eV, which corresponds to metallic Ti(O) (Figure 4a). Small contributions of titanium compounds (II, III, IV) to the Ti 2p bands in high-resolution XPS spectra (in total approximately 30%) are most likely due to the surface oxidation of Ti clusters that occurred during their transportation to XPS. Keeping the samples in ambient atmosphere leads to further oxidation and the properties of the particles become very similar to those of type 2 (oxidized by plasma immediately after the deposition). This follows from the antibacterial tests described below as well as from the optical transmittance measurements presented in Supporting Information File 1. In-flight oxidation (type 3) results in an increase of the contribution of titanium oxide compounds. Namely, the band of TiO_2_ (IV) becomes the most intense one in the spectra (Figure 4b). The fraction of metallic compound is found to be less than 5%.

### Antibacterial tests

**Figure 5:**
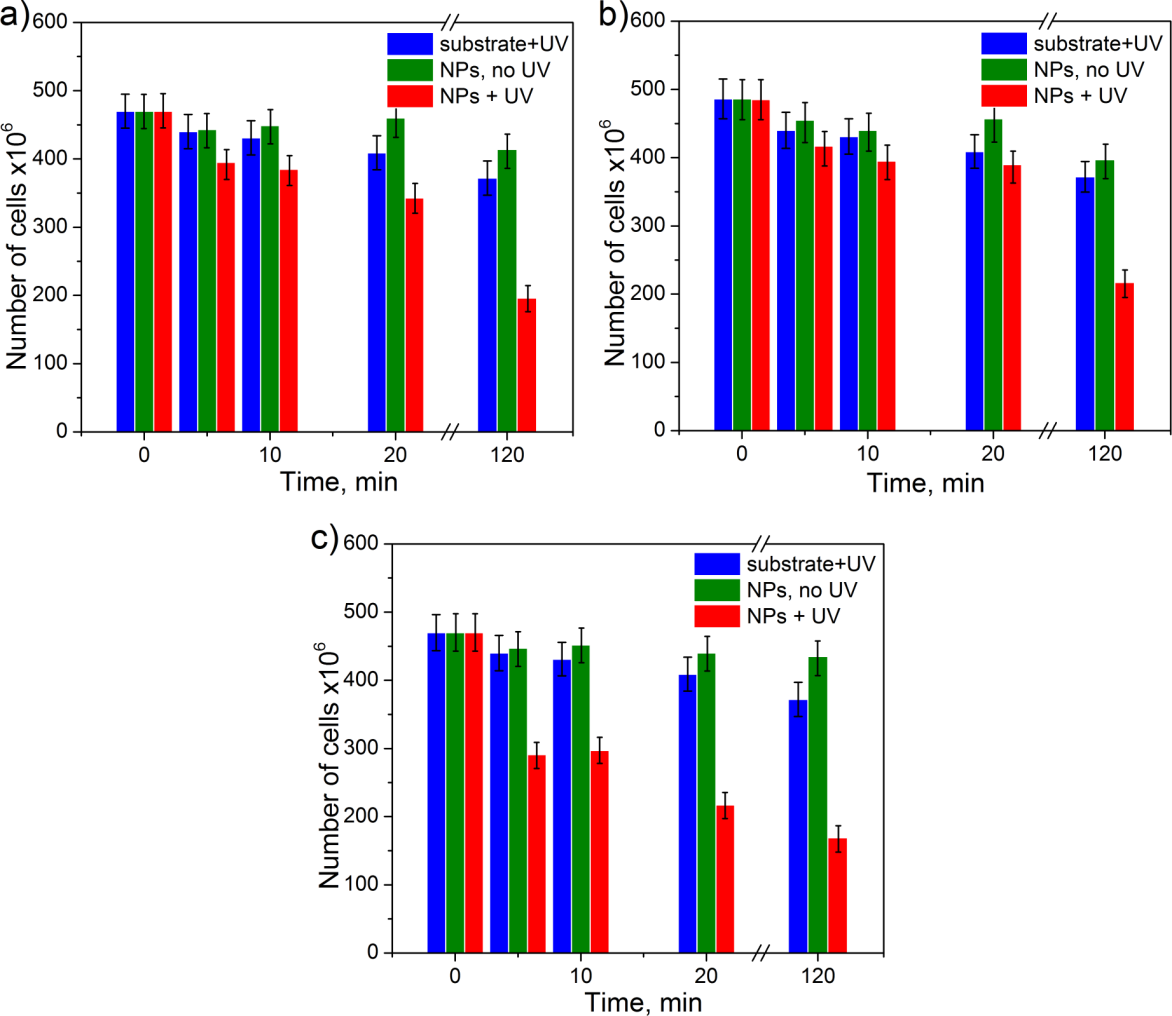
Evolution of cell number with time on the samples of (a) type 1, (b) type 2 and (c) type 3 without and with UV illumination. Zero time corresponds to the initial number of bacteria incubated on the samples. Efficiency in destroying the bacteria by UV light on substrates without particles is also shown for comparison. Vertical bars illustrate typical uncertainty in the bacteria counting.

The results of the antibacterial tests for the samples of type 1 are shown in Figure 5a. It can be seen that the amount of live cells is reduced by about 10–15% during the first 10 min upon UV illumination. Longer exposures of up to 120 min lead to a significant decrease of viability. The survival ratio (number of live cells after 120 min with respect to that immediately after the deposition) is found to be around 0.42 for the first cycle (see Table 1). The relatively long initial time for inactivation of the bacteria can be related to the hydrophobicity of the PS surface with NPs preventing a fast contact with *E.coli*. Another explanation is in possibility of membrane repair and recovery of the damaged bacteria at an earlier stage. It was shown in [16–17] that ROS attack the outer cell membrane and first cause its decomposition, while bacterial dysfunction and death only occur under longer UV illumination. For longer interaction times one should also consider the increasing coverage of NPs by dead cells, which impedes affecting the live cells. Thus, one can expect some kind of saturation in the antibacterial efficiency of our samples. UV light without the presence of NPs kills bacteria much less efficiently yielding a survival ratio of 0.77–0.79 after 120 min (see Figure 5). Antibacterial efficiency of the samples with NPs but no UV illumination is very small showing a survival ratio of 0.84–0.93 (Figure 5).

**Table 1:** Survival ratio of *E.coli* cells deposited on TiO*_x_* and Cu NPs after 120 min. In the case of TiO*_x_* the samples were exposed to UV light.

sample	TiO*_x_* type 1	TiO*_x_* type 2	TiO*_x_* type 3	Cu

1st cycle	0.46	0.48	0.37	0.32
2nd cycle	0.56	0.58	0.28	0.47
3rd cycle	0.55	0.56	0.27	0.57
4th cycle	0.48	0.49	0.30	0.62
5th cycle	0.55	0.60	0.32	0.63
6th cycle	0.59	0.56	0.38	0.68
7th cycle	0.57	0.60	0.39	0.70

The dynamics of the antibacterial effect of type-2 samples is very similar to that of type-1 samples, as one can see in Figure 5b. This agrees well with the analysis of properties of both samples presented above. Samples of type 3 demonstrate the highest antibacterial efficiency (Figure 5c). The survival ratio is reduced to 0.65 within the first 5 min and further decreases to 0.37 after 120 min. One of the factors for the high efficiency can be related to the structure and composition of the particles, in particular, to the high fraction of titanium dioxide as found by XPS. Another reason could be the aggregation of NPs leading to relatively large 3D structures that increase the overall surface area of TiO*_x_* available to produce more ROS and leading to a more efficient killing of the bacteria.

The samples demonstrate a good repeatability of antibacterial efficiency after a number of cycles. The results of survival ratio of *E.coli* cells for seven sequential cycles and every type of sample are shown in Table 1. It can be seen that the ratio is stable within approximately ±15% from cycle to cycle. The deviations can be related to small changes in the experimental conditions, for example, variations of temperature and humidity, which are difficult to keep exactly the same and which can affect the growth of bacteria. Nevertheless, one can conclude that efficiency in destroying the bacteria for all three types of TiO*_x_* do not degrade and the samples can be reused a number of times.

**Figure 6:**
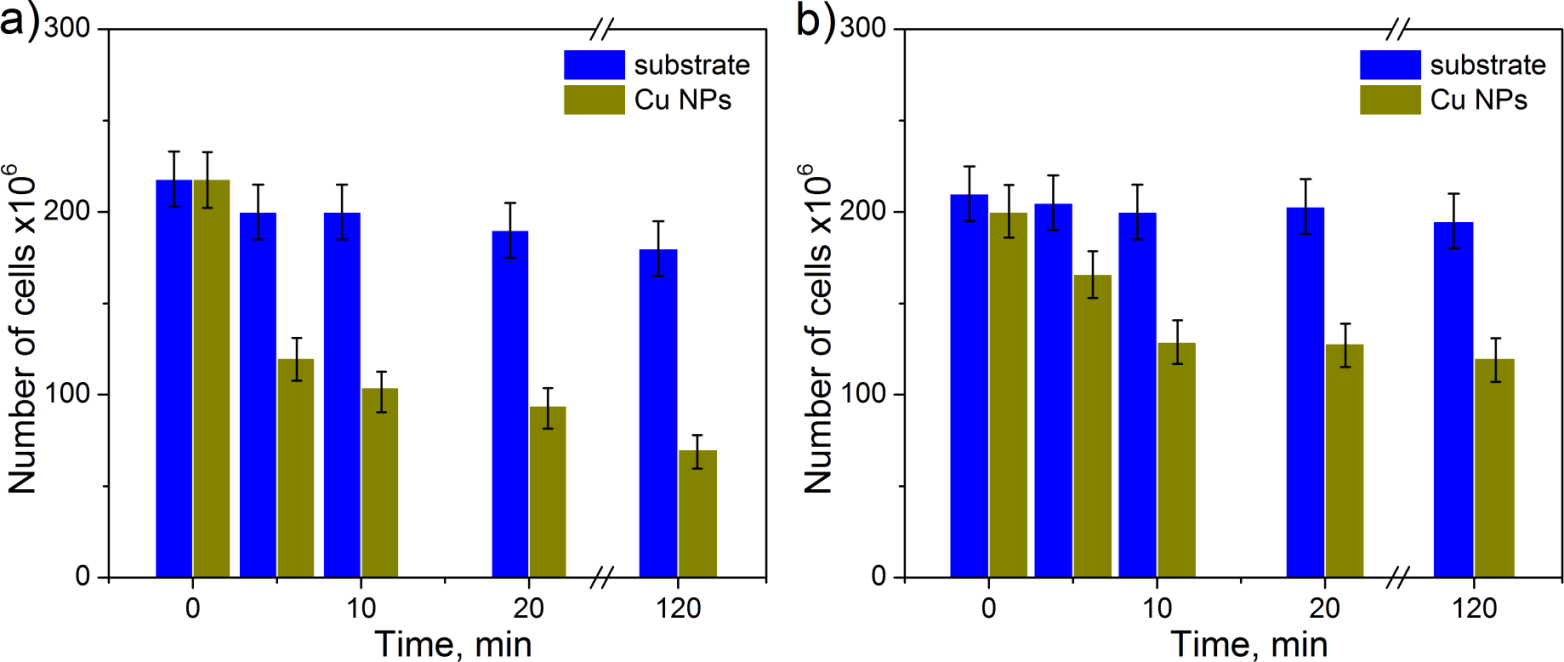
Evolution of cell number with time on the sample with Cu NPs during (a) the first and (b) the third cycle. The change in number of bacteria on substrates without particles is also presented for comparison showing the deviations within the counting uncertainties illustrated by vertical bars.

The results of antibacterial test of Cu NPs on PS are presented in Figure 6. In the first antibacterial cycle, the decrease in number of live bacteria at the initial stage of the test is faster compared to the case of TiO*_x_* particles (compare Figure 6a with Figures 5a,b). This is related to the different mechanism of destroying the cells compared to the semiconductor particles. TiO*_x_* NPs, in general, need UV activation to be antibacterial, since they do not have a direct antibacterial effect, but their mode of action is through the generation of ROS that have a negative effect on the viability of microorganisms. This implies that the generation of a sufficiently high concentration of ROS around the nanoparticles depends on the irradiation time and availability of water molecules around the nanoparticles. Furthermore, the ROS need to pass through the bacterial membrane to develop their full capacity. In comparison, the metallic NPs, such as Ag or Cu, show a direct antibacterial effect. Furthermore, Cu NPs under ambient conditions can easily lead to the formation of CuO, which also displays a direct toxicity for microorganisms [18]. They already disturb microorganisms when being in contact with the bacterial surface by reacting with proteins forming pores in the membrane and perturbing the membrane potential leading to cell death. Furthermore, ions can be transported through the membrane into the cell and by replacing the original ions disturb proteins in the cytosol. Taken all these processes into consideration, metallic NPs with direct toxicity are expected to act faster in killing bacteria than the NPs needing an external activator. And this is confirmed by the initial stage of the tests.

After 120 min the survival ratio for the films with Cu NPs is decreased to around 0.32, which is very similar to that of type-3 samples with TiO*_x_* NPs. However, contrary to titanium oxide, the bactericidal efficiency of Cu NPs gradually decreases with the number of cycles as can be seen in Table 1. After seven cycles, the survival ratio reaches approximately 0.7. Increase of the exposure time to 180 min in the 7th cycle helps to decrease the survival ratio to around 0.60. Since the AFM measurements do not show any considerable decrease in the particle surface coverage with increased number of cycles, the reduction of antibacterial efficiency of Cu NPs can be assigned to a degradation of metallic copper and conversion to copper compounds due to the long-time exposure to environmental conditions and to the solutions used for bacteria deposition and the following washing. An investigation of these mechanisms requires an additional more detailed study.

Finally, it would be of practical interest to evaluate the antibacterial efficiency of our approach compared to standard approaches. The disinfecting effect of TiO*_x_* has been studied so far mainly in solutions. One of the examples presented in [19] also studies the efficiency in destroying *E.coli* since it is an indicator bacterium for drinking water. The advantage of TiO*_x_* NPs suspended in solution is the larger contact area of the particles with the microorganisms leading to a much better bactericidal efficiency. However, the studies presented in this review showed that the TiO*_x_* suspensions under UV irradiation need to be active up to 120 min to provide the highest efficiency [19]. In our studies, we have seen that the highest efficiency of the TiO*_x_* particles also requires incubation for 120 min. These results suggest that the NPs deposited on the substrate surface show their activity in the similar way like the dissolved species. However, the disadvantage of the surface-immobilized particles is that only surface films can be treated but not liquid volumes. On the other hand, the reusability of NPs is much more important than their activity. The same immobilized TiO*_x_* particles have been used up to seven times and still show a stable reduction of the number of microorganisms. This is a huge advantage over the NPs dispersed in solution, which can typically be used only once.

Another big advantage of the proposed method is the partial embedding of NPs into polymer films. The copper NPs used in our experiments show their highest activity already after 120 min while in the case of metal–polymer composites in which the NPs are mixed into the medium volume, like Cu NPs embedded in polypropylene films [20], the activation requires a twice longer time. However, the efficiency in killing the bacteria was higher in that case. Unfortunately, we are not able to compare the reusability of our PS films with Cu NPs with those tested in [20], because such experiments were not carried out. However, we would like to stress that despite the considerable reduction of the activity already after the 3rd cycle in our case the particles still exhibit antibacterial properties, which are slowly degrading up to the 7th cycle.

## Conclusion

Antibacterial coatings were prepared by deposition of Ti and Cu nanoparticles from cluster beams on polystyrene films followed by thermal annealing above the glass transition temperature. Ti clusters are oxidized in three different ways: by long-term exposure to ambient atmosphere, by plasma-enhanced oxidation directly after the deposition and by in-flight oxidation prior the deposition on substrates. The annealing favours partial embedding of the NPs into polymer films thus ensuring a high resistance against wash-out in procedures involving liquids. Controlling the deposition time gives a possibility to tune the surface coverage of NPs for optimisation of the coating properties. Polymer film as a host material provides flexibility and plasticity to form the composites in required shapes.

The tests with *E.coli* show the antibacterial efficiency of composites with both types of NPs. Since the mechanisms of deactivating the bacteria are different for semiconducting TiO*_x_* and metallic Cu, the dynamics of bactericidal efficiency is also found to be different. Samples with TiO*_x_* NPs demonstrate a very good capability to be reused a number of times. Among them, the samples of type 3 (in-flight oxidation of Ti clusters) show the best bactericidal efficiency. The efficiency of all samples does not degrade during long-term storage (over 6 months) after the preparation. However, these composites need UV illumination complicating the application to some exent.

The use of the polymer films with Cu NPs as bactericidal agents does not require UV light but the use leads to gradual decrease of efficiency. On the other hand, these coatings would be cheaper and easier to apply. Comparison of bactericidal efficiency is found to be lower for our supported NPs compared to standard tests using NPs in solutions. Thus, more studies are required to improve this important parameter. However, the advantages of the proposed method are in the reusability of the samples and in the possibility to use polymer composites for the cases when solutions are not possible. One more important issue in relation to the use of copper should be considered, namely a potential contamination with Cu ions. Hence, polymers with Cu NPs should be used carefully in the cases of food or drugs.

## Experimental

### Preparation of polymer films with metal clusters

PS films are prepared by a standard spin-coating procedure from a solvent mixture of 2 wt % PS in toluene. Thin films are coated on silicon and quartz substrates of 10 × 10 mm^2^ and annealed at 110 °C (above the glass transition temperature). The thickness of PS films was measured by ellipsometry and found to be 50 ± 5 nm.

**Figure 7:**
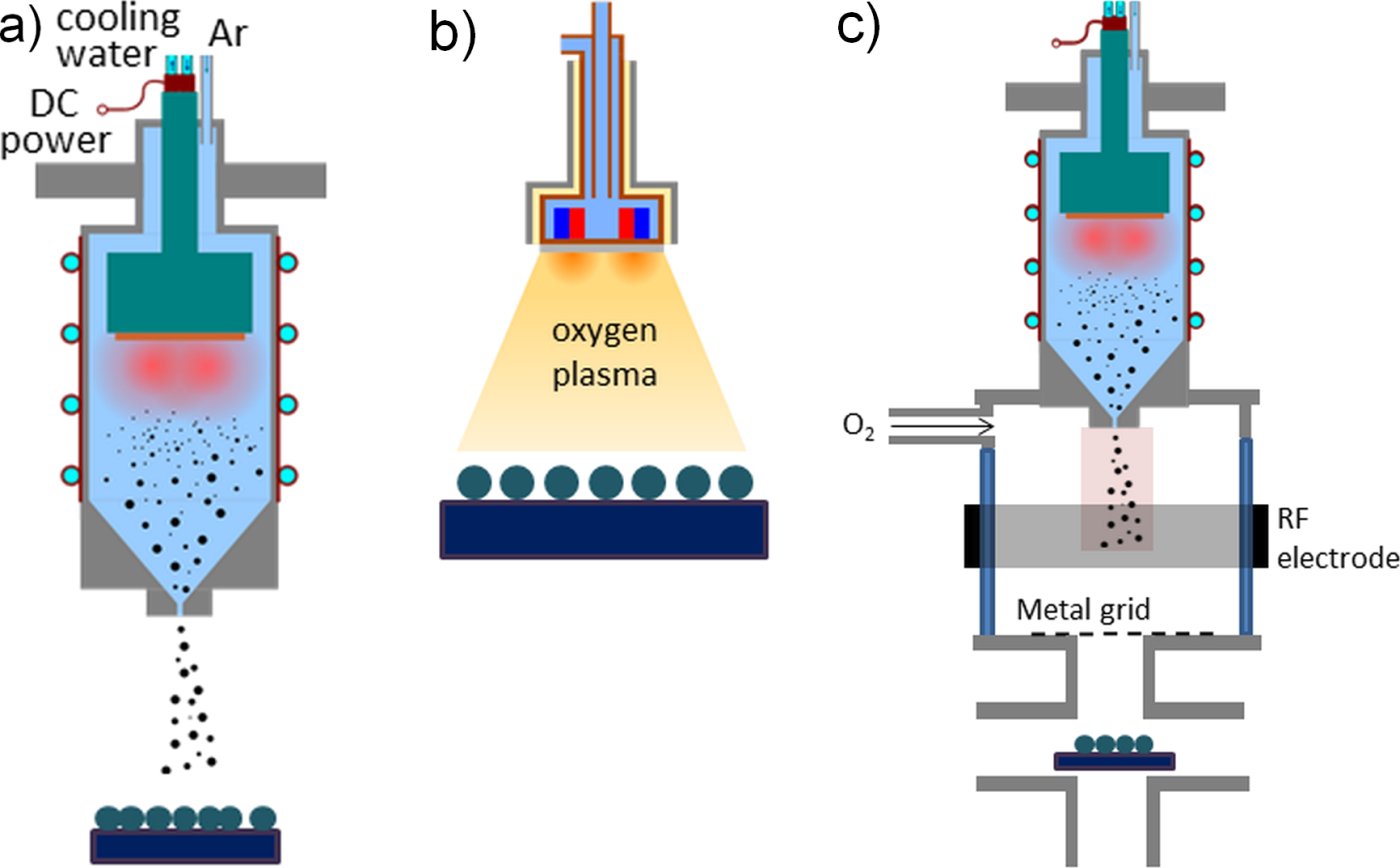
Schematic picture of (a) cluster deposition for samples of type 1, (b) treatment with oxygen plasma for samples of type 2 and (c) in-flight oxidation of the clusters for samples of type 3.

Ti clusters are produced by magnetron sputtering in a cluster source similar to the one described elsewhere [21]. This source is based on planar, water-cooled DC magnetron equipped with Ti target (99.99% of purity) inserted into a water-cooled aggregation chamber. The chamber is separated from the rest of the deposition system by a conical lid with 1.5 mm orifice. After the expansion through this orifice the clusters are deposited on a substrate as schematically shown in Figure 7a. Produced clusters are either left untreated (referred in the paper as “type 1”) and simply deposited on the substrates or treated in two different manners. For “type 2”, the deposited particles are exposed to an RF capacitively coupled oxygen plasma (Figure 7b). Oxygen plasma treatment is performed at a pressure of 4 Pa, an applied RF power of 40 W and at a distance between RF electrode and a sample of 4 cm. For “type 3”, the clusters are in-flight plasma-treated prior to the deposition on a substrate. In this case the plasma is ignited in an auxiliary cylindrical glass chamber (10 cm in diameter) with an RF electrode powered from the outside positioned in between the output orifice of the source and the substrate holder (Figure 7c). Oxygen is introduced at flow of 0.8 sccm and the treatment is performed at a pressure of 1 Pa and an applied power of 10 W.

Deposition of Cu clusters is carried out using magnetron sputtering cluster apparatus (MaSCA) [15]. MaSCA utilizes a commercial source (NC200U, Oxford Applied Research) where a metal target of 99.99% purity is sputtered, clusters are formed and expanded into the source chamber through the nozzle with a 3 mm orifice. Thereafter, they are collimated into a beam by the conical-shaped skimmer and steered into the electrostatic quadrupole mass selector. The clusters in the source are formed in different sizes; a significant fraction of them is ionized, which allows for mass (size) selection by electrostatic fields as earlier described in [22]. For the current experiments particles of mean diameter of approximately 15 nm are used.

**Figure 8:**
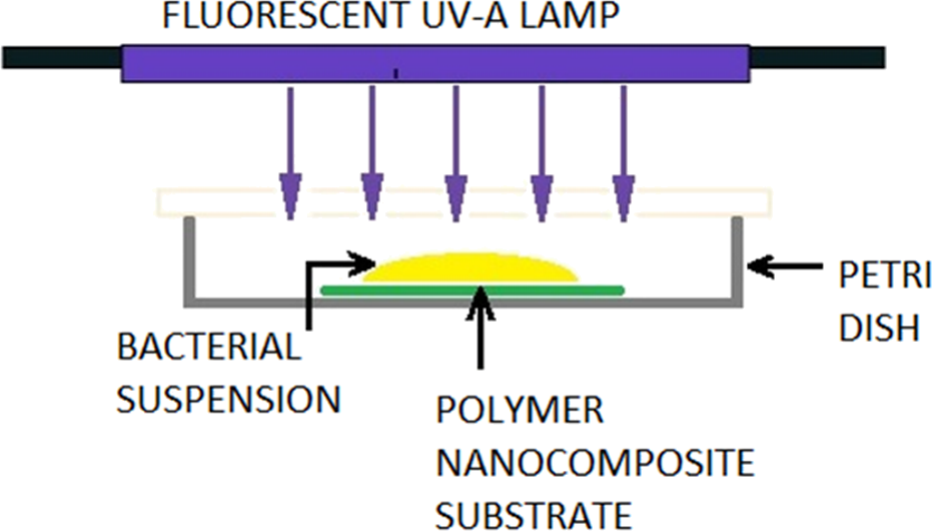
Schematic picture of setup for antibacterial experiments with TiO*_x_* NPs.

To improve the adhesion of the deposited NPs, the samples with deposited clusters are annealed at 120 °C for 5 min in ambient atmosphere. As shown elsewhere [14–15], the annealing promotes partial immersion of metal NPs into polymer films and, thus, significantly enhances the resistance against removing the particles from the surface during the wet procedures applied in the following tests. For testing the antibacterial properties of composite films with Ti/TiO*_x_* NPs a simple setup has been constructed allowing for the illumination of the samples by UV light (λ = 320–380 nm) as shown in Figure 8. *E.coli* bacteria are used for the tests.

### Characterization techniques

The surface morphology of the samples with supported NPs is studied by AFM in tapping mode using Ntegra Aura nanolaboratory (from NT-MDT). Commercial cantilevers with sharp silicon tips (radius of curvature <10 nm, force constant of 3.5–6.0 N/m) are used. SEM analysis is carried out by means of Mira3 (Tescan) microscope operated with accelerating voltage of 30 kV in secondary electron mode.

The chemical composition of the NPs is determined by means of XPS. The spectra are recorded by the spectrometer equipped with a hemispherical analyzer (Phoibos 100, Specs) and a line delay detector with five channels. The XPS scans are acquired at constant take-off angle of 90° using an Al Kα X-rays source (1486.6 eV, 200 W, Specs). High-resolution Ti 2p spectra are recorded at pass energy of 10 eV with 10 scans (dwell time 100 ms, step 0.05 eV). Prior the analysis, XPS spectra are charge referenced to adventitious carbon peak at 285 eV. Spectral analysis is performed using CASA XPS software. For the Ti 2p peak fitting the procedure described in [23] is applied. The Ti 2p_3/2_ and Ti 2p_1/2_ are fitted with four separate peaks that correspond to metallic Ti and three titanium oxides Ti(II), Ti(III) and Ti(IV). Metallic Ti is fitted by Lorentzian asymmetric peak in contrast to titanium oxide peaks for which Gaussian/Lorentzian line shape is used.

### Bactericidal experiments

An *E.coli* culture is grown in Luria Bertani medium overnight at 37 °C. The optical density (OD) of the culture is adjusted with fresh medium and applied to the different samples. Two groups of TiO*_x_* substrates of every type are used in the experiments: one with UV illumination and one without. The substrates with Cu NP are not UV treated, neither before nor during or after the growth experiments. Samples of 50 µL have been taken after 5, 10, 20 and 120 min of incubation. Reference samples are also prepared where the bacteria are deposited on substrates without clusters and illuminated by UV light. In order to find bactericidal efficiency of the samples, a standard growth curve (OD for liquid culture of bacteria versus time) is recorded by using the OD600 method (OD measurements at wavelength of 600 nm). Additionally, the samples for each time point of OD600 measurements are plated in a dilution series on agar plates and incubated overnight at 37 °C. The following day the colonies on the plates are counted and the relation between OD600 and colony forming units is established. This method does not directly discriminate between live and dead cells but a decreasing or a stagnating number of cells mean an increasing amount of dead cells in the medium, because the number of cells should increase exponentially under conventional growth conditions. Thus, this calibration procedure allows to measure the OD and to recalculate it into the number of live bacteria for the bactericidal experiments.

Repeatability of antibacterial efficiency of every group of samples is also tested by washing out the bacteria at the end of each test (by thoroughly rinsing with deionised water) and depositing the new grown cultures on the same samples. Every sample is tested for up to 7 cycles.

## Supporting Information

File 1 Optical extinction spectra of samples with TiO*_x_* NPs.
