# Supplementary material for: Comparative study of antibacterial properties of polystyrene films with TiOx and Cu nanoparticles fabricated using cluster beam technique
Source: Beilstein J Nanotechnol. 2018 Mar 12;9:861–9. doi: 10.3762/bjnano.9.80 (PMC5870157; doi:10.3762/bjnano.9.80)
Supplement: File 1 — Optical extinction spectra of samples with TiOx NPs. [file Beilstein_J_Nanotechnol-09-861-s001.pdf]

Supporting Information

for

Comparative study of antibacterial properties of polystyrene films with TiO_x and Cu nanoparticles fabricated using cluster beam technique

Vladimir N. Popok*[1], Cesarino M. Jeppesen[1], Peter Fojan[1], Anna Kuzminova[2], Jan Hanuš[2] and Ondřej Kylián[2]

Address: [1] Department of Materials and Production, Aalborg University, Skjernvej 4A, 9220 Aalborg, Denmark and [2] Department of Macromolecular Physics, Charles University, V Holešovičkách 2, 18000 Prague 8, Czech Republic

Email: Vladimir N. Popok - vp@mp.aau.dk

* Corresponding author

Optical extinction spectra of samples with TiO_x NP

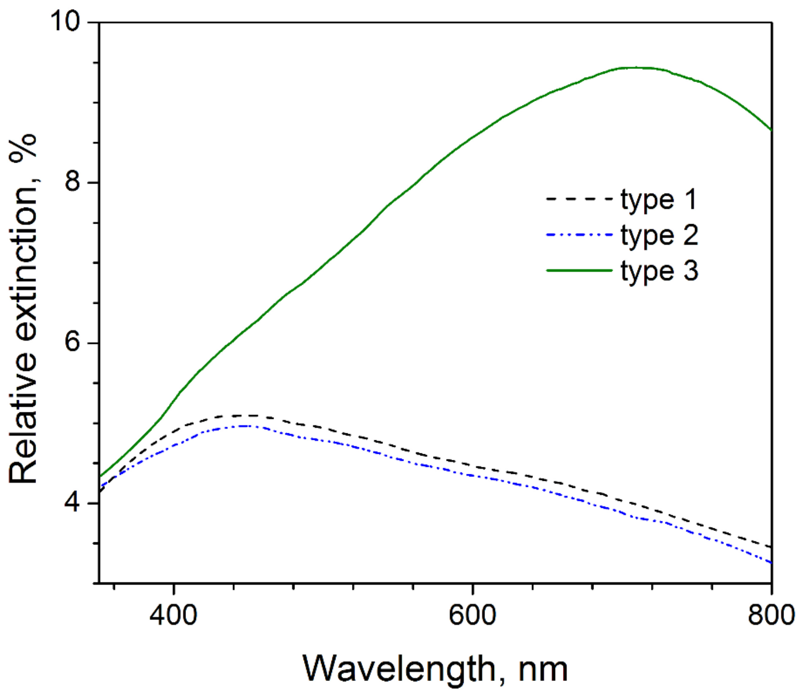

Figure S1: Extinction spectra of samples with deposited TiO_x NPs of types 1, 2 and 3. Samples of type 1 and 2 show almost identical spectra, while for type 3 the extinction is quite different.
